# Coexistence of nontuberculous mycobacterium and IgG4-related disease in a solitary pulmonary nodule: A case report

**DOI:** 10.1097/MD.0000000000018179

**Published:** 2019-11-27

**Authors:** Kyungsoo Bae, Hyo Jung An, Kyung Nyeo Jeon, Dae Hyun Song, Sung Hwan Kim, Ho Cheol Kim

**Affiliations:** a Department of Radiology, Institute of Health Sciences, Gyeongsang National University School of Medicine, Jinju; b Department of Radiology; c Department of Pathology, Gyeongsang National University Changwon Hospital, Changwon; d Department of Pathology; e Department of Thoracic Surgery; f Department of Internal medicine, Gyeongsang National University School of Medicine, Jinju, South Korea.

**Keywords:** cavity, computed tomography, IgG4-related lung diseases, lung nodule, nontuberculous mycobacterium

## Abstract

**Rationale:**

Immunoglobulin G4-related disease (IgG4-RD) is regarded as an immune-mediated systemic fibroinflammatory disease. Several studies have linked IgG4-RD to infections such as tuberculosis and actinomycosis. However, the coexistence of IgG4-RD and non-tuberculous mycobacterium (NTM) in a single pulmonary nodule has not been reported yet.

**Patient concerns:**

A 76-year-old male patient presented with cough and sputum. A solitary pulmonary nodule suspicious of lung cancer was found on chest CT.

**Diagnosis:**

Through video-assisted thoracoscopic biopsy, a diagnosis of co-existing NTM and IgG4-RD in a single nodule was made.

**Interventions:**

Antibiotic treatment was applied for pneumonia developed after surgery. The patient was also supported by extracorporeal membrane oxygenation and mechanical ventilation since his pneumonia was refractory to medical treatment.

**Outcomes:**

The patient expired on the 60th postoperative day due to multiple organ failure.

**Lessons:**

IgG4-RD can occur singularly or accompanied by other diseases. We report a solitary pulmonary nodule caused by NTM and concurrent IgG4-RD, suggesting a possible association between these 2 entities. Immunologic relations between IgG4-RD and accompanying infection should be further investigated.

## 1 Introduction

Non-tuberculous mycobacterium (NTM) pulmonary disease has been increasingly recognized in immunocompetent patients.^[1]^ When presented as a solitary pulmonary nodule or mass, it can be misdiagnosed as lung cancer or tuberculoma.^[2]^

Immunoglobulin G4-related disease (IgG4-RD) is newly defined as a fibroinflammatory process. It has been reported in various organs with different names such as Mikulicz's syndrome, Küttner's tumor, Riedel's thyroiditis, and idiopathic retroperitoneal fibrosis.^[3]^ Although this disease forms a distinct clinical entity, its pathogenesis is not clearly understood yet. Diseases occurring in association with IgG4-RD, either synchronously or metachronously, has been suggested as an etiologic trigger for abnormal IgG4-related immunological reactions.^[3]^

We report a case that highlights radiology-pathology correlation of solitary pulmonary nodule due to concurrent diagnosis of NTM and IgG4-RD that suggests a possible link between the 2 diseases.

## 2 Case presentation

This study was approved by our institutional review board. The patient's family members provided consented to publish this case. A 76-year-old male patient presented cough and sputum. He was a 30 pack-years ex-smoker. He had unstable angina and received stent insertion in right coronary artery 2 years ago. His complete blood count showed no leukocytosis (6.16 × 10^9^/L). His body temperature was 36.5°C. His blood pressure was 135/75 mmHg with pulse rate of 67/minute.

**Figure 1:**
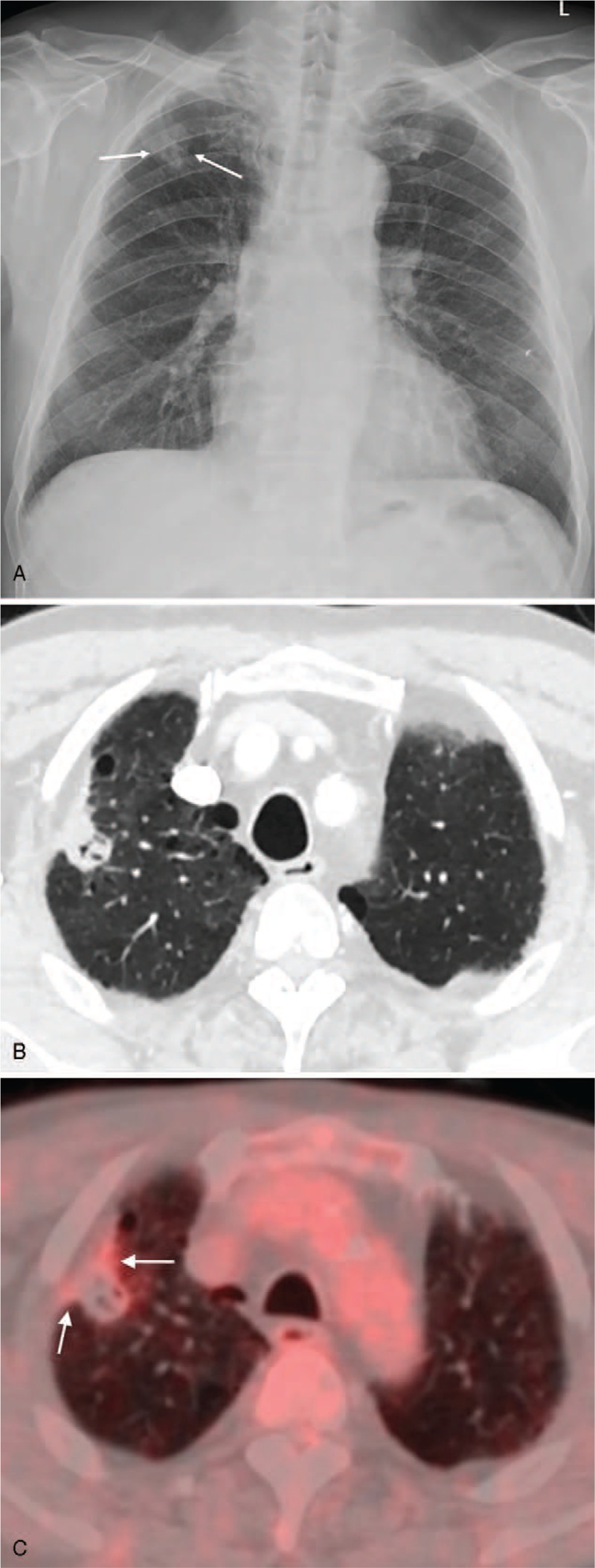
A 73-year-old male patient presented with incidental lung nodule. A. Chest X-ray showing a nodule overlapping with the 2nd rib (arrows) in the right upper lobe. B. Chest CT scan showing a cavitary nodule with eccentric wall thickening and adjacent pleural retraction. C. F-18-FDG-PET/CT scan revealing hypermetabolic activity (SUVmax = 2.8, arrows) at the pleural side of the lesion.

**Figure 2:**
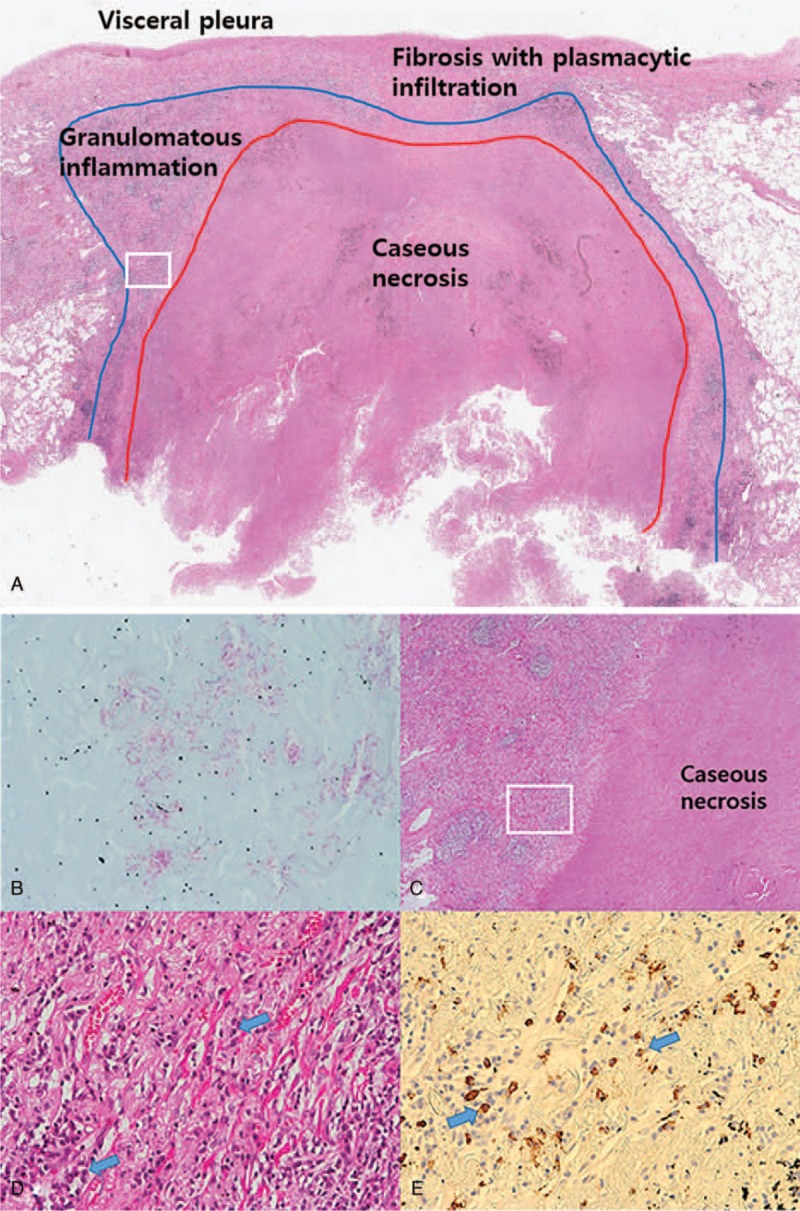
Pathologic features of the resected nodule. A. The nodule is consisted of caseous necrosis (defined by red line), surrounding granulomatous area (defined by blue line), and subpleural fibrosis with lymphoplasmacytic infiltration (×10). B. AFB stain showing numerous acid-fast bacilli in the necrotic area. (×400) Non-tuberculous mycobacterial infection was confirmed through polymerase chain reaction later. C. A magnified view showing chronic granulomatous inflammation with caseous necrosis, suggestive of tuberculosis or non-tuberculous mycobacterium (×20). Note plasma cell rich area between the caseous necrosis and the granulomatous inflammation (white box). D. A magnified view from a white box in C showing infiltrating plasma cells (arrows) in the abutting area between the caseous necrosis and the granulomatous inflammation (×200). E. Immunohistochemical staining showing positive IgG4 cells (arrows) up to 70 cells per high power field.

On chest X-ray, there was a nodule in the right upper lobe that was not detected on chest X-ray obtained 2 years before (Fig. 1A). On chest CT scan, the lesion was a 2.1 cm sized cavitary nodule located in the periphery of the right upper lobe (Fig. 1B). There were no satellite nodules or centrilobular nodules in adjacent lung. There was no significant lymphadenopathy. The possibility of lung cancer could not be excluded because the lesion showed eccentric thickening of soft tissue, extending into adjacent pleura. On F-18-FDG-PET/CT scan, hypermetabolic activity (SUVmax = 2.8) was noted at the periphery and pleural side of the lesion (Fig. 1C). Video-assisted thoracoscopic biopsy was performed for tissue confirmation. Because malignancy was suspected on frozen section of the specimen, right upper lobectomy was performed. On permanent sections of the specimen, the main portion of the nodule consisted of chronic granulomatous inflammation with caseous necrosis (Fig. 2A). In the peripheral and pleural side of the lesion, there were lymphoplasmacytic infiltration and whorled fibrosis. Numerous acid-fast bacilli were noted in the necrotic area, suggestive of *Mycobacterium tuberculosis* or NTM (Fig. 2B). Polymerase chain reaction using tissue was positive for NTM but negative for *M. tuberculosis*. There were infiltrating plasma cells in the abutting area between the caseous necrosis and the granulomatous inflammation and subpleural area (Fig. 2C). In this areas, positive IgG4 cells were noted (up to 70 cells per high power field) (Fig. 2D and E). Serum IgG4 level was elevated (657 mg/dl). Based on these results, the patient was diagnosed as having NTM and IgG4-RD in a single nodule.

On the 7th postoperative day, the patient developed pneumonia caused by *Klebsiella pneumoniae.* On the 20th postoperative day, fungal infection was superimposed. The disease progressed to adult respiratory distress syndrome. Since pneumonia was refractory to medical treatment, the patient was supported by extracorporeal membrane oxygenation and mechanical ventilation. However, the patient eventually developed multiple organ failure and expired on the 60th postoperative day.

## 3 Discussion

IgG4-RD is a recently recognized fibroinflammatory disease characterized by abundant IgG4-positive plasmacytic infiltration. When presented as a parenchymal mass or nodule, the lesion often mimics a primary lung malignancy. In the present case, IgG4-RD existed at the periphery of NTM lung disease. Eccentric wall thickening and pleural retraction on chest CT and FDG-uptake in the corresponding area on F-18-FDG-PET/CT made physicians consider a possibility of malignancy.

Solitary pulmonary nodule is a rare radiologic presentation of NTM lung disease, occurring in only 10 among 269 cases from a large tertiary center.^[2]^*M avium* or *M intracellulare* was the causative organism in most reported cases of NTM manifesting as solitary nodule.^[2,4]^ Polymerase chain reaction can differentiate between *M tuberculosis* and NTM in acid-fast bacilli-positive specimens.

The present case showed simultaneous existence of NTM and IgG4-RD in a solitary pulmonary nodule. Although pathophysiological mechanism of IgG4-RD has not been clearly defined yet, autoimmunity and infectious agents have been suggested as potential immunologic triggers.^[3]^

In type 2 helper T cell predominant immune reaction, cytokines such as interleukin (IL)-4, IL-5, IL-10, and IL-13, and transforming growth factor β can be overexpressed. These cytokines contribute to eosinophilia, elevated serum IgG4 and IgE concentrations, and progression of fibrosis that are characteristics of IgG4-RD.^[3,5]^ A study of one patient with IgG4-RD involving pancreas, liver, and salivary glands has found accumulation of IgG4-expressing plasma cells in the colon and terminal ileum.^[5]^ Peripheral blood mononuclear cells isolated from the patient showed enhanced production of IgG4 and IL-10 on stimulation with Toll-like receptor ligands.^[5]^ This raised the possibility that various species of bacteria including intestinal microflora might have induced production of IgG4 through innate immunity.

Several studies linking *M tuberculosis* and IgG4-RD in pulmonary/ extrapulmonary organs have been reported.^[6–8]^ Concurrent IgG4-related lymphadenopathy was reported in a patient with disseminated NTM infection.^[9]^ However, co-occurrence of NTM and IgG4-RD in a pulmonary lesion has not been reported before. In the present case, his immunologic response to NTM might have precipitated the development of IgG4-related lesions. On pathologic examination, fibroinflammatory tissues suggesting IgG4-RD existed at the periphery of caseation necrosis and granulomatous area, creating irregular wall thickening and pleural invasion. We postulate that NTM in this patient might have activated a type 2 helper T cell response that in turn leads to overexpression of interleukins and transforming growth factor.

Diagnosis of IgG4-RD can be made using comprehensive diagnostic criteria combined with organ-specific criteria.^[10]^ Key pathological features are dense lymphoplasmacytic infiltrate, storiform fibrosis, obliterative phlebitis, and eosinophilic infiltration. IgG4/IgG-positive cell ratio > 40% and/or >10 IgG4-positive cells/high power field are required.^[10]^ A definitive diagnosis of IgG4-RD can be made in patients who fulfill all of the following 3 criteria:

1) organ involvement,
2) a serum IgG4 concentration >135 mg/dl, and
3) histopathological findings of >10 IgG4 cells/HPF and IgG4/IgG-positive cell ratio >40%.

We presented a solitary pulmonary nodule due to NTM and concurrent IgG4-RD, suggesting a possible association between the two entities. Further investigations and more proof from clinical cases are needed to clarify the relationship between infection and IgG4-RD.

## Author contributions

**Conceptualization:** Kyung Nyeo Jeon.

**Data curation:** Kyungsoo Bae, Hyo Jung An, Kyung Nyeo Jeon, Sung Hwan Kim, Ho Cheol Kim.

**Formal analysis:** Hyo Jung An, Dae Hyun Song.

**Methodology:** Hyo Jung An, Dae Hyun Song, Ho Cheol Kim.

**Resources:** Sung Hwan Kim.

**Writing – original draft:** Kyungsoo Bae, Kyung Nyeo Jeon.

**Writing – review & editing:** Kyung Nyeo Jeon.
